# Baclofen for Spasticity in Multiple Sclerosis: A Comprehensive Narrative Review

**DOI:** 10.3390/biomedicines14102357

**Published:** 2026-10-16

**Authors:** Alan M. Palmer

**Affiliations:** Elixa MediScience Ltd., 24–28 Brockenhurst Road, Ascot, Berkshire SL5 9DL, UK; alan.palmer@elixamedisci.com

**Keywords:** baclofen, receptors, GABA-B, muscle spasticity, multiple sclerosis, injections, spinal, pharmacokinetics, downregulation, substance withdrawal syndrome

## Abstract

Baclofen, a GABA-B receptor agonist developed as an anticonvulsant in the 1960s and repurposed for spasticity in the 1970s, is recommended as a first-line oral treatment for spasticity in multiple sclerosis (MS) by UK NICE guidance and is used similarly, though not necessarily designated identically, in many other jurisdictions. It acts at two sites within the spinal stretch reflex arc: presynaptically on Ia afferent terminals in the dorsal horn, where it reduces excitatory neurotransmitter release, and postsynaptically on ventral horn interneurons and α-motoneurons, where it hyperpolarises the cell and suppresses firing. Only a small fraction of an oral dose crosses the blood–CNS barrier, so oral dosing requires high systemic doses that cause sedation, ataxia and CNS depression. Intrathecal delivery bypasses the barrier, allowing a much lower dose and fewer brain-mediated side effects. Other drawbacks include a short, variable half-life, GABA-B receptor downregulation contributing to adverse effects and intrathecal tolerance, and withdrawal that has proved fatal. The narrow efficacy-to-side-effect margin is probably intrinsic to GABA-B receptor activation. This narrative review covers baclofen’s neuroanatomy, pharmacokinetics, administration routes, efficacy, comparators, long-term outcomes, interactions, special populations, and regulatory history. Long-term follow-up shows durable control without worsening sedation, but the one study using validated quality-of-life measures found no significant improvement despite clear spasticity reduction. Weighing benefit against harm, this review concludes that baclofen offers a net benefit, but conditionally: only for carefully selected, individually titrated, and actively monitored patients. Because this is a narrative rather than systematic review, source selection was not governed by a prespecified protocol, some clinical estimates rely on secondary or aggregate data, and the underlying literature is heterogeneous in design and quality.

## 1. Introduction

Baclofen is one of the oldest drugs still in first-line clinical use for spasticity. It was synthesised in the 1960s as a candidate anti-epileptic agent, designed as a lipophilic analogue of γ-aminobutyric acid (GABA) intended to cross the blood–brain barrier more readily than GABA itself. Its anticonvulsant activity proved disappointing, but during clinical testing, it was noted to reduce muscle tone, and by the 1970s, it had been repurposed and established as an antispastic agent, particularly in multiple sclerosis (MS) and spinal cord disease [1,2]. Five decades later, it remains, together with gabapentin, the first-line oral treatment recommended for MS-related spasticity by UK NICE guidance and is one of the most widely prescribed antispasticity drugs worldwide.

The population affected is substantial. MS affects an estimated 2.9 million people worldwide [3]. Spasticity affects up to ~84% of MS patients at some point, though only around a third rate it moderate, severe, or totally limiting—the group most likely to need pharmacological treatment [4]. Swedish population-based prescribing data found that ~10% of newly diagnosed and 19% of prevalent MS patients were prescribed baclofen, with over half discontinuing within six months [5]; prescribing proportions of this kind vary substantially between countries with different guidelines, reimbursement systems, and prescribing cultures, so these Swedish figures should not be read as globally representative. Applying this country-specific ~10–20% range to the global MS population gives a purely hypothetical illustrative figure of roughly 300,000–580,000 people with MS taking baclofen worldwide at any time; this calculation is not a validated epidemiological estimate, only an order-of-magnitude illustration of scale, and should not be cited as a standalone figure.

Baclofen acts as an agonist at the GABA-B receptor, a metabotropic, G-protein-coupled receptor distinct from the ionotropic GABA-A receptor targeted by benzodiazepines. GABA is the principal inhibitory neurotransmitter of the central nervous system, and GABA-B receptor activation—presynaptically, by suppressing calcium influx and reducing the release of excitatory neurotransmitters such as glutamate and substance P, and postsynaptically, by increasing potassium conductance and hyperpolarising neurons—suppresses mono- and polysynaptic spinal reflexes [1]. This is the basis of baclofen’s antispastic action at the spinal cord, with a supraspinal contribution also thought to play a role.

## 2. Methods

The literature search for this narrative review was performed on English-language sources up to September 2026, using PubMed/MEDLINE (U.S. National Library of Medicine, National Institutes of Health, Bethesda, MD, USA), Google Scholar (Google LLC, Mountain View, CA, USA), and manufacturer prescribing information (US FDA Drugs@FDA database, Silver Spring, MD, USA; the European Medicines Agency (Amsterdam, The Netherlands); and the UK electronic Medicines Compendium, Datapharm Ltd., Leatherhead, UK). For PubMed/MEDLINE, the core search string was (“baclofen” [Title/Abstract] OR “baclofen” [MeSH Terms]) AND (“multiple sclerosis” [Title/Abstract] OR “spasticity” [Title/Abstract] OR “pharmacokinetics” [Title/Abstract] OR “intrathecal” [Title/Abstract] OR “GABA-B” [Title/Abstract] OR “withdrawal” [Title/Abstract] OR “tolerance” [Title/Abstract] OR “drug interaction” [Title/Abstract] OR “arbaclofen” [Title/Abstract]); equivalent free-text combinations were run in Google Scholar. This returned on the order of several hundred unique records after de-duplication (identified by DOI/PMID and, where absent, by title-and-author matching)—an approximate order of magnitude rather than a logged count, as no formal PRISMA-style tracking was performed (see below). Titles and abstracts were screened by the author for relevance to the themes listed above; full texts were obtained for records judged potentially relevant, and these, together with records identified by hand-searching the reference lists of retrieved reviews and by consulting manufacturer prescribing information, were assessed for inclusion in the final reference list of 115 sources. Records were excluded at screening if they were not in English, were conference abstracts superseded by a later full publication, addressed baclofen in a clinical context unrelated to MS spasticity (e.g., alcohol use disorder pharmacotherapy) without transferable relevance, or were secondary summaries adding no information beyond a primary source already captured. Where multiple sources addressed the same question, primary research reports were prioritised over narrative summaries of that same work; where evidence conflicted (for example, the opposing effects of pharmacological GABA-B activation versus genetic GABA-B downregulation on oligodendrocyte biology, Section 4.4), both findings are presented together with an explicit statement that they are not reconciled by the available data, rather than one being silently preferred. Preclinical (in vitro or animal) evidence was included where it provides mechanistic grounding for a clinical observation or where no human data exist on a given point, and it is explicitly labelled as preclinical throughout rather than presented alongside clinical findings without distinction (see Section 5.2). Literature selection was considered complete once the searches above, across all three sources, ceased to return new records judged relevant to the review’s defined scope (baclofen’s pharmacokinetics, administration routes, clinical efficacy, and long-term use in MS spasticity, together with the mechanistic, regulatory, and comparative context needed to interpret that evidence) at the September 2026 cut-off date. This is a narrative rather than a systematic review: no formal eligibility checklist, quality-scoring instrument, or risk-of-bias tool was applied, record counts in each stage were not tracked in the manner of a PRISMA flow diagram, and selection ultimately rested on the author’s judgement of relevance and contribution to the narrative; where the underlying evidence is heterogeneous or limited, this is stated explicitly in the text rather than papered over. Finally, this review’s scope is deliberately broader than the core themes named in its title. Regulatory history, intellectual property, neuroanatomy, and glial pharmacology are included not as independent topics but rather because each is needed to interpret those core themes: the regulatory and patent landscape (Section 3) explains why intrathecal and extended-release formulations have developed as they have, and the receptor neuroanatomy (Section 4) underpins the pharmacokinetic and tolerance discussions that follow (Section 5 and Section 9).

## 3. Regulatory Approval History and Intellectual Property

Baclofen was synthesised in 1962 by the Swiss chemist Heinrich Keberle at Ciba-Geigy (formed in 1970 from the merger of Ciba AG and J.R. Geigy SA; now part of Novartis following the 1996 Ciba-Geigy/Sandoz merger), as one of a series of β-aryl-halogenated GABA derivatives designed to cross the blood–brain barrier more readily than GABA itself [6,7]. Its intended anticonvulsant application failed, but its muscle-relaxant properties, noted incidentally during clinical testing, were pursued instead. Baclofen was registered in Europe as Lioresal^®^ roughly a decade after its synthesis [7] and reached the United States market in 1977 under NDA 020075 [8]; this gives the compound a regulatory history now approaching sixty years.

Key regulatory approvals for baclofen, by agency and formulation, are summarised in Table 1.

The intellectual property picture reflects this history. Baclofen’s original composition-of-matter patent expired decades ago; the molecule is off-patent and genericised, which explains its low cost and inclusion on national essential medicines lists worldwide [9]. Commercial activity has accordingly shifted to formulation- and delivery-specific patents—Gablofen^®^ (intrathecal, CNS Therapeutics/Mallinckrodt), Lyvispah™ (oral granules, Saol Therapeutics), and a US patent family addressing batch-to-batch metabolite variability [12]—all covering an off-patent active ingredient. The one genuinely novel chemical entity is arbaclofen (Section 6.3): its extended-release formulation (Ontinua^®^ ER, Osmotica/RVL Pharmaceuticals) has FDA orphan drug designation and reported patent protection to 2036 [13], though it had not yet received FDA approval as of the most recent filings reviewed [14].

A related caveat applies to Table 1 itself: as its caption notes, the earliest European and UK approval dates are approximate reconstructions from historical secondary sources rather than primary regulatory filings, as original 1970s licensing records are not readily available online. These entries should be read as indicative of approximate timing rather than as verified historical fact; it is the overall sequence and pattern of approvals, rather than the precise early dates, that should be relied upon.

### Structural Relationship to GABA

Baclofen’s chemical structure makes its origin as a rationally designed GABA derivative directly visible (Figure 1). Both molecules share an identical four-carbon backbone running from a primary amine to a terminal carboxylic acid; baclofen differs only in carrying a 4-chlorophenyl substituent at the β-carbon (C3) of that backbone. This single lipophilic addition is what allows a pharmacologically meaningful, if still small, fraction of an administered dose to cross the blood–brain barrier and reach CNS GABA-B receptors (Section 5.1)—GABA itself, lacking this substituent, does not cross the blood–brain barrier in clinically useful amounts, which is precisely why the original 1960s medicinal chemistry programme that produced baclofen was undertaken in the first place [6,7].

## 4. Neuroanatomy and Mechanism of Action

### 4.1. Receptor Structure and Signalling

The GABA-B receptor is a metabotropic, class C G-protein-coupled receptor that, unlike most GPCRs, functions only as an obligate heterodimer: GABA-B1 carries the extracellular GABA/baclofen-binding domain, while GABA-B2 traffics the complex to the surface and couples it to its intracellular Gi/Go effector [15,16]. Activation produces two location-dependent effects. Presynaptically, Gβγ subunits inhibit voltage-gated calcium channels at the axon terminal, reducing the release of glutamate, substance P or GABA itself, and inhibit adenylyl cyclase [16]. Postsynaptically, the same signalling opens GIRK/Kir3 potassium channels, producing a slow hyperpolarisation that reduces firing probability [16,17]. This dual action—damping transmitter release at one synapse while hyperpolarising the next neuron—underlies GABA-B-mediated inhibition throughout the CNS, and both mechanisms operate together at baclofen’s principal therapeutic site.

Baclofen’s affinity for the GABA-B receptor is, in absolute terms, modest rather than high. Radioligand binding at cloned human GABA-B(1a,2) and GABA-B(1b,2) heterodimers gives an IC50 of approximately 30–32 µM—similar to GABA itself—with a Ki around 6 µM [18,19]. Functional agonist assays report a tighter apparent potency, IC50/EC50 35–180 nM [19,20], a discrepancy most likely reflecting receptor reserve and signal amplification rather than a true affinity difference. This matters for the route comparison in Section 6.2: intrathecal delivery achieves local CSF concentrations in the low-micromolar range, comfortably within this window, whereas oral dosing produces CSF concentrations roughly an order of magnitude lower (Section 5.1)—helping explain why oral dosing struggles to achieve full receptor engagement without raising systemic exposure towards sedative levels.

### 4.2. Spinal Localisation and the Antispastic Mechanism

Spasticity is a core feature of the upper motor neuron syndrome and is classically defined as a velocity-dependent increase in tonic stretch reflexes with exaggerated tendon jerks, resulting from hyperexcitability of the stretch reflex. Under normal conditions, descending corticospinal and, in particular, reticulospinal and vestibulospinal pathways continuously regulate the excitability of segmental spinal circuits, including the gain of the stretch reflex and the threshold for motoneuron firing. Damage to these descending pathways—as in MS, spinal cord injury, or stroke—removes this regulatory drive and disinhibits the underlying spinal circuitry rather than creating a new pathological pathway; spasticity is therefore best understood as normal spinal reflex machinery operating without its usual supraspinal restraint.

The core anatomical substrate is the stretch reflex arc. Sensory Ia afferent fibres from muscle spindles make monosynaptic excitatory contact with alpha motoneurons in the ventral horn; the same afferents also drive polysynaptic pathways, including reciprocal inhibition and flexor reflex circuits, contributing to the flexor/extensor spasms seen alongside tonic hypertonia. GABA-B receptors act at two independent points within this arc. Presynaptically, on central terminals of Ia and other primary afferents in the dorsal horn, activation suppresses glutamate and substance P release onto interneurons and motoneurons [21]; recent work confirms this action directly and shows that it is measurably reduced after spinal cord injury, identifying loss of this endogenous brake as a plausible contributor to spasticity itself, which baclofen then restores pharmacologically [22]. Postsynaptically, GABA-B receptors on ventral horn interneurons and motoneurons open GIRK channels and hyperpolarise the cell, a second, independent layer of inhibition at the reflex output.

A further postsynaptic mechanism is relevant to the phasic, self-sustaining spasms and clonus that trouble many patients as much as tonic stiffness. Spinal motoneurons possess voltage-gated, largely L-type calcium channels that generate persistent inward currents (PICs): once triggered, PICs amplify and prolong the motoneuron’s response, producing self-sustained firing from a brief trigger. PIC amplitude is normally kept in check by descending monoaminergic input; after spinal cord injury, this regulation is disrupted and PICs become hyperexcitable, which generates the prolonged spasms of spinal cord-mediated spasticity. Recordings in spinal-injured animals show that baclofen suppresses these abnormal PICs—and the monosynaptic reflex itself—at submicromolar concentrations via the same GIRK-mediated hyperpolarisation [23]. This gives baclofen a mechanistic basis for treating spasms and clonus distinct from, and additive to, its action on the tonic stretch reflex (Figure 2). Together, this combined presynaptic-plus-postsynaptic, tonic-plus-phasic action is why baclofen treats both tonic hypertonia and phasic spasms and why intrathecal delivery achieves a larger antispastic effect for a given systemic exposure than the oral route (Section 6.2).

Figure 2 summarises this arc and baclofen’s dual site of action within it.

### 4.3. Supraspinal Localisation and the Anatomical Basis of Off-Target Effects

GABA-B receptors are not confined to the spinal cord. Immunohistochemical mapping shows GABA-B1/GABA-B2 subunits distributed throughout the neuraxis, with particularly high density in the thalamus, neocortex, hippocampus, habenula, and brainstem monoaminergic nuclei, in addition to the cerebellum [24]. This widespread supraspinal distribution directly explains baclofen’s principal dose-limiting adverse effects (Section 11): a graded consequence of the drug’s own target being present outside the spinal cord, not a separate receptor being engaged. In the cerebellum, dense postsynaptic expression on Purkinje cell dendritic spines is the most direct anatomical correlate of baclofen-induced ataxia and dose-dependent rotarod impairment near the antispastic range (Section 15) [17,25]. In the thalamus/neocortex, activation contributes to sedation and cognitive slowing; in the hippocampus, to confusion and memory effects; and in brainstem nuclei, to the bradycardia, hypotension and respiratory depression of severe toxicity (Section 11). Table 2 summarises these locations and their clinical correlates.

The location, pre-/postsynaptic site of action and clinical correlate of the GABA-B receptor populations discussed in this review are summarised in Table 2.

### 4.4. Glial GABA-B Receptors and a Potential Remyelinating Action (Preclinical Evidence)

The receptor biology described above is neuron-centred, but GABA-B receptors are not confined to neurons: a growing, largely preclinical literature shows they are also expressed on astrocytes, microglia and cells of the oligodendrocyte lineage, where they contribute to excitatory/inhibitory balance, synaptic pruning and myelination, and have been implicated in the pathobiology of several CNS disorders, MS among them [26]. This glial dimension is mechanistically distinct from the spinal antispastic action described in Section 4.2 and is not an established part of baclofen’s clinical effect, but it is a genuine and active area of investigation, summarised here for completeness.

Astrocytes express both GABA-A and GABA-B receptors as well as GABA transporters, which makes them “GABAceptive” cells that buffer extracellular GABA (and, in some contexts, release it), thereby shaping neuron–glia and glia–glia signalling relevant to the excitatory/inhibitory balance discussed throughout this review [27]. Microglia are similarly GABAceptive: in vitro, baclofen dose-dependently attenuates Toll-like receptor 3/4-driven pro-inflammatory cytokine release from murine glia and from human peripheral blood mononuclear cells, though this anti-inflammatory effect was blunted in cells from patients with relapsing–remitting MS compared with healthy donors, alongside reduced GABA-B receptor mRNA expression in the patient cells—raising the possibility that baclofen’s immunomodulatory potential may itself be attenuated in the disease it is most often prescribed for [28].

The most developed line of evidence concerns oligodendrocyte progenitor cells (OPCs) and myelination. OPCs and Schwann cell precursors express functional GABA-B receptors that, in vitro, promote OPC proliferation, migration and differentiation and increase myelin protein expression when activated by baclofen specifically [29]. Building on this, baclofen given to adult mice after lysolecithin-induced demyelination increased myelin basic protein and myelin-associated glycoprotein levels and enhanced OPC differentiation and remyelination, an effect attributed to greater myelin output from existing oligodendrocytes rather than a larger OPC pool [30]. Unpublished doctoral work from the same group extended this to experimental autoimmune encephalomyelitis (EAE), a model that better reflects MS’s autoimmune character, reporting that baclofen also ameliorated EAE severity and increased OPC differentiation within lesions.

This encouraging picture is complicated by more recent, cell-type-specific work: genetic downregulation of GABA-B receptors specifically in OPCs, rather than pharmacological activation, was found to protect against demyelination in the cuprizone and lysolecithin models—the opposite direction of effect from the baclofen studies above [31]. The likely explanation is that de- and remyelinating phases, and cell-autonomous versus organism-wide receptor manipulation, are not pharmacologically equivalent, but this also means that the net effect of baclofen on human demyelinating disease cannot be safely inferred from either result alone. All of this evidence comes from rodent and ex vivo models; no clinical trial has tested whether baclofen exerts a disease-modifying effect in patients with MS, and the CNS concentrations achieved by antispastic dosing (Section 5 and Section 6) have not been validated against those used experimentally. This glial mechanism should therefore be read as a hypothesis-generating rationale for drug repurposing rather than an established clinical benefit, and it does not alter the benefit–risk assessment developed in Section 16.

To make the clinical-translation boundary explicit: every finding described in this subsection—the in vitro cytokine and OPC data, the lysolecithin and EAE remyelination studies, and the cell-type-specific knockdown findings—derives from rodent or ex vivo systems, not from patients, and none has been tested at the doses or treatment durations used clinically for spasticity. This body of work should therefore be read as a plausible preclinical signal that warrants dedicated clinical investigation, not as evidence that baclofen has, or should be expected to have, a disease-modifying effect in people with MS.

## 5. Pharmacokinetics

### 5.1. Absorption and Distribution

Oral baclofen is rapidly and well absorbed from the gastrointestinal tract, with an absolute bioavailability generally reported between 70% and 85% [32,33]; crossover pharmacokinetic studies in healthy volunteers have reported means of 74% and 80%, respectively [34,35]. Peak plasma concentrations are reached quickly, typically within 2–3 h of an oral dose [33]. Protein binding is low, at around 30%, and the apparent volume of distribution is small (roughly 0.7–2.5 L/kg depending on the study) [36], consistent with a hydrophilic molecule that nonetheless crosses the blood–brain barrier sufficiently to reach its CNS target—though, as discussed in Section 5.3, this crossing is a rate-limiting step for the drug’s action.

Direct evidence for just how restricted this brain penetration is comes from a rat brain microdialysis study measuring baclofen simultaneously in hippocampal interstitial fluid (ISF), whole-brain tissue, CSF and plasma following intravenous bolus and constant infusion [37]. At pseudo-steady state, the concentrations in ISF, whole brain and CSF were all ~30-fold lower than that in unbound plasma—direct quantitative confirmation of the restricted CNS distribution inferred elsewhere in this review. Kinetic modelling showed that this was not simply a passive permeability limit: the influx clearance from plasma to brain (CLin, 0.00157 ± 0.00076 mL/min/g) was ~40-fold lower than the efflux clearance back to plasma (CLout, 0.0712 ± 0.0529 mL/min/g), and efflux was substantially reduced by co-administration of probenecid, an organic anion transport inhibitor. Together, these findings indicate that baclofen’s limited brain penetration reflects an efficient, transporter-mediated efflux system at the blood–brain barrier rather than a simple failure to cross passively—this is directly relevant to Section 5.3: because efflux, not influx, is rate-limiting, oral dose increases yield, diminishing the incremental antispastic benefit relative to the accompanying rise in systemic exposure.

Expressed in molar terms, chronic oral dosing in the standard 30–80 mg/day range produces steady-state plasma concentrations of roughly 0.47–1.87 µM, the reference interval used by clinical laboratories for baclofen plasma-level monitoring [38]. It is this systemic exposure that must increase to control spasticity, as only a small, transporter-limited fraction reaches the CSF (above); correspondingly, systemic adverse effects are common across this dose range—a 2017 evidence-based review estimated the overall incidence of oral baclofen adverse effects (sedation, weakness, dizziness, confusion) at 25–75% of patients, consistent with figures as low as 10% reported elsewhere in the literature [39] and with the 63%-versus-36%-on-placebo sedation figure already noted in Section 11.1.

### 5.2. Elimination and Half-Life

Baclofen undergoes minimal hepatic metabolism (on the order of 15% of a dose, mainly by deamination); the majority (roughly 70–85%) is excreted unchanged by the kidney [36], which makes renal function the principal determinant of clearance and a key consideration in older patients or those with renal impairment (Section 14.1).

The elimination half-life is short and variable between studies and individuals—reported values cluster around 2.5–6 h [33,36], from about 2.3–3.4 h in children [40] up to 5.2–5.8 h in healthy adults [35] and 6.8 h in one combined plasma-and-urine modelling study [41]. This inter-individual variability, layered on an already short half-life, is clinically important: the interval over which a patient’s blood level remains above the concentration needed to control spasticity varies considerably from person to person, so a dosing interval that suits one patient may leave another with a considerable trough. In overdose, the half-life can be dramatically prolonged (case reports describe values up to 34–36 h) [33], reflecting saturation of renal clearance mechanisms at high plasma concentrations.

Because baclofen’s clearance is so dominated by the kidney, several pharmacokinetic studies have specifically measured urinary, not only plasma, drug concentrations. A combined plasma-and-urine modelling study in four healthy volunteers given a single 40 mg oral dose found a mean of 69% (SD 14) of the dose recovered unchanged in urine, a renal excretion rate constant of 0.35 (SD 0.24) h^−1^, and an apparent renal clearance essentially equal to creatinine clearance, with no dependence on urine flow rate or pH—indicating that baclofen is cleared largely by glomerular filtration, with little net contribution from active tubular secretion or reabsorption [41]. This is consistent with the renal impairment dosing guidance in Section 14.1. Other groups have developed dedicated plasma-and-urine HPLC assays to quantify baclofen and its inactive deaminated metabolite in urine [42,43,44], but, in practice, this has remained principally a research and bioanalytical-validation tool rather than a routine clinical test; plasma-level monitoring (Section 5.3) is the more established approach.

To avoid conflating findings from different species when interpreting the pharmacokinetic data above, Table 3 separates this evidence by whether it derives from animal (preclinical) or human (clinical) studies and states explicitly where a parameter has not been directly measured, using a comparable methodology, in both.

### 5.3. The Narrow Therapeutic Window

Baclofen’s central limitation as an antispastic drug is pharmacokinetic rather than pharmacodynamic: because only a small fraction of an oral dose penetrates the CNS, the plasma (and, hence, CNS) concentrations required to achieve a useful reduction in spasticity sit close to those that cause sedation, dizziness, and cognitive slowing. This is compounded by its short and variable half-life: patients must take the drug frequently—commonly three to four times daily and, in some patients, up to five or six times a day—to maintain plasma levels within this narrow effective range without troughing into a symptomatic “wearing-off” period before the next dose, in which spasticity, spasms and stiffness re-emerge.

Wearing-off is particularly troublesome overnight, where it interacts with the well-described diurnal (day–night) variation in spasticity: tone and spasm frequency are characteristically at their worst in the early hours of the morning (around 4–6 a.m.). A late-evening baclofen dose that has largely worn off by this time leaves patients exposed to the coincidence of a physiological trough in the drug and a physiological peak in spasticity, which is frequently reported as the most uncomfortable and sleep-disrupting period of the 24 h cycle. This has been a major driver of interest in longer-acting formulations (Section 6.3) and in intrathecal delivery, which bypasses much of the peripheral pharmacokinetic variability altogether.

## 6. Modes of Administration

### 6.1. Oral Baclofen

Oral tablets and oral solutions are the default first-line formulation. Treatment is started low and titrated slowly—a typical regimen begins at 5 mg two to three times daily, increasing by 5–15 mg every three days as tolerated, to a usual maintenance range of 30–80 mg/day in divided doses (three to four times daily) [45]; the FDA-approved maximum is 80 mg/day, although higher doses (up to 100–150 mg/day) have been used under specialist supervision in refractory cases. If no benefit is seen at the maximum tolerated dose within about six weeks, NICE guidance recommends stopping rather than persisting [46].

### 6.2. Intrathecal Baclofen (ITB)

For patients with severe spasticity refractory to, or intolerant of, oral therapy, baclofen can be delivered directly into the cerebrospinal fluid (CSF) via an implanted, programmable pump and intrathecal catheter, most commonly sited in the lumbar subarachnoid space. This route achieves therapeutic CSF concentrations at doses roughly 100-fold lower than the equivalent oral dose, because the drug is delivered directly to its site of action rather than relying on systemic absorption and blood–brain barrier penetration—substantially reducing systemic (supraspinal and peripheral) side effects such as sedation for a given antispastic effect.

Patients are first screened with a single lumbar bolus (typically starting at 50 micrograms, with a second, larger bolus if there is no response) to confirm a positive response before committing to pump implantation [45]. Maintenance infusion doses are highly individualised, typically in the range of 90–700 micrograms/day, with limited experience above 1000–1200 micrograms/day [45]. CSF clearance after a bolus dose is rapid, with a reported CSF elimination half-life of only around 1.5 h [36]—one reason why continuous infusion, rather than intermittent bolus dosing, is used for chronic maintenance therapy.

Because the drug is delivered directly into CSF rather than absorbed and redistributed via plasma, systemic exposure on intrathecal therapy is correspondingly minimal: concurrent plasma concentrations during intrathecal infusion are typically ≤47 nM, roughly 10- to 40-fold lower than the 0.47–1.87 µM steady-state range seen with oral dosing (Section 5.1) and consistent with the manufacturer’s own characterisation of plasma levels as low (0–23 nM) during intrathecal infusion [47]. This roughly order-of-magnitude-lower systemic exposure is the direct pharmacokinetic basis for intrathecal therapy’s markedly lower burden of sedation, cognitive dulling and generalised weakness relative to oral dosing at an antispastic-equivalent effect and is a principal reason why patients with oral-refractory or oral-intolerant spasticity are transitioned to a pump [48,49].

Physically, the intrathecal component is a soft, radio-opaque silicone catheter (external diameter roughly 1–2 mm, internal lumen ~0.5 mm) introduced into the lumbar subarachnoid space through a Tuohy needle and threaded cephalad under fluoroscopic guidance. Placement has traditionally favoured the low thoracic region (T10–T12) for lower-limb-predominant spasticity, but catheters are sited anywhere from the lumbar sac to the upper cervical spine—including, when lumbar access is precluded by prior fusion or scarring, retrograde placement via a foramen magnum or lateral C1–C2 approach—depending on which body regions need coverage [50]. Rather than a single open end, the distal portion is typically capped and fitted with several (commonly three) small lateral side holes, so the drug is released along a short column rather than from one point source. This softens, but does not eliminate, the point source assumption behind the gradients described below and is also the anatomical basis for an inflammatory granuloma that can form at the catheter tip, where local concentration is chronically highest [51,52].

Drug movement within the intrathecal space itself is not a simple craniocaudal bulk flow. Pharmacokinetic modelling in patients receiving graded intrathecal bolus doses confirms a steep baclofen concentration gradient along the spinal axis, with the drug remaining preferentially concentrated around the catheter tip rather than mixing uniformly through the CSF column [53]. Published gradients are steep: cisternal CSF concentrations run at roughly a quarter of simultaneous lumbar concentrations [54], and concentrations measured with increasing distance from a T10 catheter tip fall to around 30% at 5 cm, 12% at 10 cm, and as little as 0.7% at the cerebral (cisternal/ventricular) level [53]. In practical terms, only a small fraction of a percent of the tip concentration reaches the cerebral ventricles from a standard low thoracic or lumbar catheter—the principal reason why supraspinal adverse effects remain uncommon at standard doses (Section 11). Non-human primate tracer studies similarly show that catheter-tip level materially determines rostral distribution, with higher, more cervically placed catheters producing substantially greater brain exposure than lower thoracic placement [55]. This diffusion- and pulsatile-mixing-dominated pattern underlies the clinical importance of catheter-tip placement. Baclofen ultimately re-enters circulation predominantly via absorption across the arachnoid villi and spinal capillary bed rather than through any directional CSF outflow tract.

This steep, distance-dependent gradient is also the direct rationale for deliberately higher catheter placement in patients whose spasticity is predominantly upper-limb or generalised, because a low thoracic or lumbar catheter delivers only a small fraction of its local concentration to the cervical cord. A systematic review found that cervical placement improved upper-limb spasticity and function without increasing complication rates relative to thoracolumbar placement, albeit on generally low-quality evidence [50]. Separately, because the therapy depends on some ongoing rostral CSF movement, an under-recognised cause of late treatment failure is rostral CSF flow obstruction—typically arachnoid scarring above the catheter tip, which can follow trauma, infection, or the granuloma noted above—confirmed on CSF flow imaging and, in reported cases, treatable by microsurgical adhesiolysis or an intradural bypass shunt [56]. Catheter-tip granuloma and rostral flow obstruction together illustrate that the fate of intrathecal baclofen depends as much on the local mechanics of CSF movement as on the drug’s own pharmacokinetics.

This favourable systemic profile is counterbalanced by a risk profile that is qualitatively different from, not simply smaller than, that of oral therapy. Because efficacy now depends on a correctly functioning implanted device rather than a swallowed tablet, intrathecal therapy substitutes lower systemic exposure for a small but serious risk of device-related error: overdose from a programming or refill error, or unintended rostral spread, can reproduce the sedation, hypotonia and respiratory depression of oral overdose (Section 11), while pump failure, catheter dislodgement, or an unrecognised empty reservoir can precipitate the same life-threatening intrathecal withdrawal syndrome described in Section 12—hyperthermia, rebound spasticity, rhabdomyolysis and multi-organ failure—which oral baclofen reinstatement does not reliably rescue once established [57].

### 6.3. Emerging and Adjunct Routes

Because conventional racemic baclofen’s frequent dosing schedule is itself a source of poor adherence and wearing-off (Section 5.3), drug development has focused on isolating its active R-enantiomer, arbaclofen, from the inactive or near-inactive S-enantiomer that makes up the other half of every racemic dose. Arbaclofen shows 100- to 1000-fold greater specificity for the GABA-B receptor than the S-enantiomer and roughly five-fold greater potency than racemic baclofen [58,59]—a clear pharmacological rationale: if only the R-enantiomer drives the antispastic effect, removing the S-enantiomer should, in principle, allow the same efficacy at lower total exposure, potentially widening the margin discussed in Section 15. An extended-release (ER) formulation, dosed twice daily using osmotic pump technology, was developed on this basis; because its pharmacokinetics differ from the R-enantiomer as delivered within racemic tablets (attributed to bioavailability rather than enantiomer interaction), there is no simple fixed equivalence between an arbaclofen ER dose and the R-enantiomer content of a racemic dose [58].

The only published randomised trial to compare arbaclofen ER directly against racemic baclofen rather than placebo alone found their antispastic efficacy statistically indistinguishable: arbaclofen ER 20 mg twice daily and baclofen tablets 20 mg four times daily produced similar improvements on the Total Numeric-transformed Modified Ashworth Scale, Clinician Global Impression of Change, and MS Spasticity Scale, with neither shown to be superior [60]. Tolerability differed more clearly: treatment-emergent adverse events occurred in 57.3% on arbaclofen ER versus 72.6% on baclofen and 50.0% on placebo, with drowsiness and dizziness less frequent on arbaclofen ER, and a significant Epworth Sleepiness Scale increase was seen with baclofen but not arbaclofen ER [60]. The trial evidence to date thus points to a tolerability advantage rather than a demonstrated efficacy advantage—though this head-to-head comparison confounds two things at once: removal of the S-enantiomer and the switch from an immediate-release, four-times-daily formulation to a smoother, twice-daily one. It cannot, by itself, separate a genuine stereochemical advantage from a purely pharmacokinetic one.

Longer-term, open-label safety data (up to 80 mg/day for one year) found at least one treatment-emergent adverse event in 86.1% of patients—most commonly urinary tract disorder (34.7%), nausea (21.7%), muscle weakness (23.8%), asthenia (18.9%), dizziness (16.1%) and somnolence (12.7%)—with 14.9% discontinuing because of adverse events, mostly muscle weakness, MS relapse, asthenia and nausea; one death (myocardial infarction) was judged unlikely to be treatment-related [59]. The relatively modest somnolence rate (12.7%) in this open-label cohort is notable set against the 63%-versus-36%-on-placebo drowsiness figure for racemic oral baclofen from the original 1977 trial (Section 11.1) [61,62], though the two figures come from different trial designs, dosing regimens and eras and are not a controlled comparison.

Weighing this evidence, arbaclofen’s risk/benefit profile is best read as plausibly, but not yet demonstrably, superior to that of racemic baclofen. The mechanistic case for a wider margin is sound (Section 15), the one head-to-head trial shows meaningfully fewer adverse events for equivalent efficacy, and long-term open-label safety data are broadly reassuring. Against this, its efficacy has not been shown to exceed that of racemic baclofen, the tolerability signal cannot be cleanly separated from the change in dosing frequency, and the compound has already failed one FDA review cycle, with no confirmed timeline for the next. For now, arbaclofen remains a plausible future alternative for patients whose main difficulty is tolerability or dosing frequency, rather than an established one.

Intravenous baclofen has also been studied, largely as a bridging strategy when oral therapy is temporarily interrupted (e.g., perioperatively), but has no established role in the routine long-term management of MS spasticity.

## 7. Clinical Efficacy in Multiple Sclerosis

Baclofen’s antispastic efficacy in MS has been demonstrated across oral and intrathecal routes, in double-blind placebo-controlled trials and in large long-term observational cohorts. Outcomes are most often reported using the Ashworth Scale (0–4) or its Modified revision (MAS: 0, 1, 1+, 2, 3, 4), the Penn Spasm Frequency Scale (0–4), the Patient-Specific Functional Scale (0–10), and visual analogue/numeric rating scales (0–10, or 0–100 mm) for pain, stiffness or global symptom burden. Table 4 summarises key studies spanning the drug’s evidence base, from its 1977 multicentre registration trial through to large modern intrathecal cohorts and a recent systematic review.

Two points are worth drawing out. First, the oral evidence base is dominated by older trials (1970s–1980s) that reported significance but not always a quantified percentage change by modern standards—a reflection of the outcome measures available at the time rather than a weakness of the drug’s effect. Second, the intrathecal literature consistently shows large relative reductions in Ashworth/MAS scores (of the order of 30–70%), generally larger than those typically reported for oral therapy in refractory patients, consistent with the much higher CSF concentrations achievable without dose-limiting CNS side-effects.

## 8. Comparative Efficacy and Place in Therapy

Baclofen does not act in isolation, and a clinically oriented review should situate it against the oral and adjunct alternatives available for MS spasticity. Gabapentin is the principal oral comparator; NICE guidance places baclofen and gabapentin on an equal first-line footing, selected according to contraindications and preference, with switching recommended if the first is not tolerated [46]. Despite its structural resemblance to GABA, gabapentin does not act at GABA receptors; its antispastic effect is thought to arise from binding the α2δ subunit of voltage-gated calcium channels, reducing calcium influx and excitatory neurotransmitter release—a mechanism distinct from baclofen’s direct GABA-B agonism. A systematic review covering baclofen, tizanidine, dantrolene and diazepam concluded that trial evidence for these four shows ‘limited evidence of effectiveness’ individually but that they are ‘approximately equally effective’ to one another; a Cochrane review of the same four similarly found insufficient evidence for firm comparative recommendations [66]. The four act through distinct mechanisms: tizanidine is a central α2-adrenergic agonist that reduces excitatory neurotransmitter release onto spinal motoneurons via a noradrenergic, rather than GABAergic, pathway; dantrolene acts peripherally, on skeletal muscle itself, by inhibiting ryanodine receptor-mediated calcium release from the sarcoplasmic reticulum and uncoupling excitation–contraction coupling—the only one of the four that does not act on the CNS; and diazepam, a benzodiazepine, is a positive allosteric modulator at the ionotropic GABA-A receptor, enhancing chloride-channel-mediated inhibition throughout the CNS, mechanistically complementary to, but distinct from, baclofen’s metabotropic GABA-B action (Section 13). In practice, the choice among these agents is usually driven by the side-effect profile (sedation and hepatotoxicity risk with tizanidine and dantrolene, respectively) rather than demonstrated superior efficacy.

Nabiximols (a tetrahydrocannabinol/cannabidiol oromucosal spray) is licensed in several jurisdictions as an add-on therapy for MS spasticity not adequately controlled by other antispasticity medication, including baclofen; observational data support meaningful improvement in spasticity-associated symptoms when added to existing regimens, including baclofen, in clinical practice [67]. Botulinum toxin injection is reserved for focal spasticity (e.g., isolated adductor or ankle plantarflexor spasticity) rather than the generalised spasticity for which baclofen is typically used, and the two are often complementary rather than competing options. Taken together, baclofen’s place in therapy is as the default first-line oral agent, escalating to intrathecal delivery for severe, refractory, or generalised spasticity, with nabiximols or botulinum toxin as adjuncts for specific residual symptom patterns rather than as baclofen replacements.

Table 5 summarises these comparators alongside baclofen itself, drawing on the systematic review and guideline evidence cited above rather than on mechanistic reasoning alone, to make the practical distinctions discussed in this section easier to scan.

Baclofen is also not intended to replace physical management, and guidelines (including NICE) recommend combining pharmacological treatment with physiotherapy—stretching, positioning, and exercise—rather than relying on medication alone [46]. The evidence for what this adds to spasticity control specifically, versus general comfort and function, is more equivocal than the near-universal recommendation suggests: an early controlled crossover trial found that baclofen alone significantly improved quadriceps spasticity, and a fully powered 2024 RCT found structured stretching and education “not essential” for managing MS spasticity impact versus a range-of-motion control, despite years of recommendation resting largely on expert opinion [68]. Massage has an even thinner evidence base and is best regarded as comfort-focused. Physiotherapy remains core to comprehensive management—particularly for range of motion and contracture prevention—but its independent contribution to spasticity control itself is less established than routine practice implies.

## 9. Effect of Chronic Use on GABA-B Receptor Number and Function

A pharmacological limitation of baclofen, distinct from its short half-life, is that continuous GABA-B receptor stimulation drives adaptive changes that blunt the drug’s own effect over time—the molecular basis of clinically observed tolerance. In rodents given repeated baclofen (for example, 5 mg/kg three times daily for five days), GABA-B receptor-stimulated G-protein activation (measured by [35S]GTPγS binding) was significantly reduced in the prefrontal cortex, septum, amygdala and parabrachial nucleus compared with untreated controls, which indicates functional desensitisation of the receptor rather than a change in the drug’s pharmacokinetics [69].

With more prolonged dosing, this desensitisation is accompanied by a genuine reduction in receptor number: repeated subcutaneous baclofen for 14 days in rats reduced radioligand binding to GABA-B receptors in the brain, together with reductions in GABA-B1 and GABA-B2 subunit mRNA and protein—consistent with true receptor downregulation (reduced synthesis and/or enhanced degradation) rather than desensitisation alone [70]. Comparable findings of reduced GABA-B receptor density have been reported after at least 21 days of baclofen pretreatment in other rodent models.

It is worth being explicit about the inferential step from the preceding paragraph to the clinic. The receptor desensitisation and downregulation data above come entirely from rodent dosing studies, which use doses, routes, and durations that do not map precisely onto human intrathecal or oral regimens. The clinical tolerance phenomena described immediately below—for example, the approximately 5% of intrathecal patients who become refractory to further dose increases—are therefore best understood as consistent with, and mechanistically plausible given, the animal receptor-adaptation data, rather than as a directly demonstrated, quantified consequence of a specific degree of human receptor downregulation, which has not itself been measured in patients.

This receptor-level adaptation has a direct clinical correlate. With chronic intrathecal baclofen, the Lioresal Intrathecal SmPC notes that approximately 5% of patients on long-term treatment become refractory to further dose increases—attributed to tolerance and, in some cases, spinal GABA-B receptor downregulation—managed by a planned dose reduction (a “drug holiday”, typically 2–4 weeks) with temporary substitution of an alternative agent (e.g., intrathecal preservative-free morphine) before resuming baclofen, often restoring responsiveness [71]. The same adaptation also has a safety implication in the opposite direction: if intrathecal delivery is abruptly interrupted (e.g., pump or catheter failure) in a patient with downregulated spinal receptors, oral baclofen substitution is often inadequate to prevent withdrawal, because spinal sensitivity to GABA-B agonism has been reset to a higher baseline requirement.

## 10. Long-Term Clinical Outcomes: Maintenance of Efficacy, Tolerance, and Quality of Life

The preceding sections establish that baclofen works and describe the receptor-level basis of tolerance (Section 9). The clinically more pressing question for a lifelong condition like MS is different: over years rather than weeks, does the benefit hold up, does the side-effect burden grow alongside it, and does controlling spasticity actually translate into a better quality of life? The answer differs markedly between oral and intrathecal use and is more nuanced than either a simple success or failure story.

In unselected, real-world oral use, persistence is poor: the Swedish population-based cohort already discussed (Section 1) found that more than half of patients discontinued baclofen within six months [5], for reasons the registry data cannot fully resolve but which plausibly include inadequate effect, intolerable sedation, or both. Intrathecal delivery is used specifically in patients for whom this oral trial has already failed, and the long-term evidence here is correspondingly richer and more reassuring, though still mixed.

Dose trajectory over time is the most direct window onto tolerance in practice. In a mixed-aetiology cohort followed for up to ten years, ITB dose rose over 18 months then stabilised around 350 micrograms/day; only 22% met a strict tolerance definition, with no clinical predictor identified [72]. This “rises-then-plateaus” pattern is not the whole picture: cohorts followed to 7–10 years, particularly MS-specific ones, show continued escalation—one 10-year series rose roughly four-fold (83 to 321 micrograms/day) [73]; a separate 7–10-year MS/SCI cohort reported increases of 157–194% [74]. Whether this reflects true tolerance, disease progression, or ongoing re-titration is not resolved by observational data. A minority experience a tolerance severe enough to require formal management: one case describes dose increases over 16 years eventually failing entirely, resolved only by a slow taper, with spasticity returning to pretreatment baseline but no worse [75]. Where dosing was instead capped deliberately, one Japanese cohort sustained control to a median of almost seven years on a comparatively low dose, with tolerance in only 5.9% [76], which suggests that some long-term dose creep may be avoidable rather than pharmacologically inevitable.

Long-term (multi-year) outcome studies of intrathecal baclofen, including dose trajectory and patient-reported outcomes, are summarised in Table 6.

Two findings in Table 6 are worth holding in tension. First, on whether long-term dose escalation buys symptom control at the cost of accumulating sedation, the >10-year follow-up data are reassuring: sleepiness was normal, fatigue only low-to-moderate, and life satisfaction correlated positively, not negatively, with dose [77]—though this needs a survivorship caveat, because patients who discontinued early from poor efficacy or intolerable side effects are structurally absent from such surveys, so the picture likely overstates how well the average new patient will do. Second, and more sobering, is the disconnect between spasticity-specific outcomes and broader quality of life: the one prospective multicentre study to formally assess validated, generic instruments (EQ-5D, SIP-68, and FIM) found only small, non-significant improvements despite clear spasticity reduction [78]. Durable control of tone, spasms and pain is realistic and largely sustained in properly selected patients, but this symptom-level success does not reliably translate into the broader disability, independence, or quality-of-life gains patients and clinicians might hope for.

This disconnect has a direct practical implication. Ashworth/MAS scores, and even the dose trajectory and tolerance data summarised in Table 6, capture what is easiest to measure—muscle tone—rather than what patients and clinicians ultimately care about: pain, function, independence, and participation in daily life. A patient, or a cohort, can show a clean reduction in tone while showing little or no improvement on EQ-5D, SIP-68 or FIM, as in the one prospective cohort to use all three validated instruments [78]. This argues for building a validated, generic quality-of-life or participation measure into the routine long-term follow-up of intrathecal baclofen, alongside tone scales rather than in place of them, so that treatment response is judged against outcomes patients would recognise as meaningful; it also argues for discussing this distinction explicitly with patients when setting expectations for therapy. Durable control of spasticity is a realistic and valuable goal in its own right, but it should not be presented to patients as synonymous with an equivalent gain in overall quality of life.

## 11. Adverse Effects and Off-Target Pharmacology

Baclofen’s adverse-effect profile is broad, and much of it reflects a loss of the spinal selectivity that underlies its therapeutic action rather than a distinct “off-target” mechanism. At therapeutic concentrations, baclofen acts predominantly on spinal GABA-B receptors; in overdose, or with reduced clearance, this selectivity is lost and supraspinal receptors are increasingly engaged, producing encephalopathy, respiratory depression, and cardiovascular instability rather than a graded extension of the antispastic effect [79]. A 2026 systematic review pooling 1618 individuals with reported oral baclofen toxicity or withdrawal found that toxicity most commonly presented as CNS depression (68%), seizures (36%), and respiratory depression (21%), particularly at doses ≥ 300 mg; management required mechanical ventilation in ~54.5% of cases, but full clinical recovery occurred in 97.7%, with low mortality (~0–4%) [80].

### 11.1. Neuropsychiatric Effects

The most common adverse effect is dose-related sedation: in the original 1977 multicentre trial, transient drowsiness was reported in 63% of patients receiving baclofen versus 36% on placebo [61,62]. Confusion, dizziness, and ataxia are similarly common and dose-related. Less common but clinically important neuropsychiatric effects include hallucinations, personality change, depression, and euphoria (the latter contributing to a recognised, if limited, potential for misuse); suicidal ideation and suicide-related events have been reported and are flagged in current product labelling [62]. Acute psychosis has been described following baclofen overdose and should be considered in the differential of new-onset psychosis in a patient known to be taking the drug [81,82]. A pharmacovigilance analysis of the FDA Adverse Event Reporting System similarly identified somnolence, confusion, seizures, and coma as principal neurological adverse events [83].

### 11.2. Seizure Threshold

Baclofen’s relationship with seizure threshold is paradoxical and depends on direction of dose change rather than a single fixed effect. Despite its origin as a failed anticonvulsant, seizures (including myoclonic seizures and non-convulsive status epilepticus) are a recognised feature of both baclofen toxicity/overdose and of abrupt withdrawal, and abrupt discontinuation may cause the loss of previously stable seizure control in patients with epilepsy; patients with a seizure history are generally considered to need closer monitoring, including EEG where clinically indicated, during initiation, dose changes, and discontinuation [33,84].

### 11.3. Cardiovascular and Respiratory Effects

Cardiovascular adverse effects include decreased cardiac output, hypotension (occasionally with orthostatic symptoms), bradycardia, and, less commonly, arrhythmias and palpitations [62,85]. Blood pressure effects are, notably, bidirectional rather than uniformly hypotensive: hypertension is a recognised feature of both the withdrawal syndrome (Section 12, where it contributes to the clinical overlap with autonomic dysreflexia) and, less intuitively, of overdose itself—one case series of baclofen overdose presentations found hypertension in 13 of 23 patients, compared with hypotension in only one, indicating that clinicians should not assume that normal or elevated blood pressure excludes significant baclofen toxicity [86]. Respiratory depression is dose-related and is the principal cause of the ventilatory support many severe toxicity cases require; it is compounded substantially by co-administration of other CNS/respiratory depressants (Section 13).

### 11.4. Other Systemic Effects

Gastrointestinal effects (nausea, constipation, and dry mouth), genitourinary effects (urinary retention or, conversely, enuresis, and rarely priapism, which may develop or recur specifically on interruption of intrathecal treatment), and ocular effects (nystagmus, blurred vision, and accommodation disturbance) are all recognised, generally dose-related, and usually reversible with dose reduction [62,85]. Long-term use has also been associated with persistent cognitive slowing, mood change, and muscle weakness in some patients, though high-quality long-term comparative data on these chronic effects remain limited.

### 11.5. Bladder and Lower Urinary Tract Effects

The genitourinary effects noted above (Section 11.4) are not simply a passive consequence of baclofen’s renal elimination; they reflect a direct pharmacodynamic action, as GABA-B receptors are present within the spinal micturition reflex circuitry itself, at the same dorsal horn/sacral level implicated in the antispastic mechanism (Section 4.2). In anaesthetised rats, systemic baclofen dose-dependently suppresses both spinal and supraspinal components of the micturition reflex, reducing detrusor contraction strength while increasing bladder capacity [87,88]; intrathecally, it reduces non-voiding bladder contractions and suppresses detrusor–sphincter dyssynergia in a rat SCI model, an effect blocked by the GABA-B antagonist saclofen—which confirms that it is receptor-mediated rather than a non-specific consequence of sedation [89].

This mechanism has both a therapeutic and an adverse-effect face, and which predominates depends on baseline bladder physiology. Baclofen was used off-label for neurogenic detrusor–sphincter dyssynergia in spinal cord injury as early as 1980, which predates its use for limb spasticity [90], and an early human study confirmed a favourable effect on detrusor hyperreflexia [91]. A more recent case series in patients receiving intrathecal baclofen after traumatic brain injury found corresponding urodynamic improvement after pump implantation—increased bladder capacity, reduced detrusor pressure, and fewer incontinence episodes—while cautioning that these effects are incidental to the spasticity indication and that urological status should be reassessed once therapy begins, because the same GABA-B-mediated suppression of detrusor contractility can just as readily produce urinary retention in a patient without overactivity [92]. This dual potential is the mechanistic basis for the urinary retention and enuresis already noted as recognised adverse effects (Section 11.4).

This pharmacodynamic action on the bladder is distinct from, though clinically compounded by, the pharmacokinetic point made in Section 5.2 and Section 14.1: because baclofen’s clearance depends almost entirely on glomerular filtration, any reduction in renal function raises systemic drug exposure for a given dose, increasing occupancy at spinal GABA-B receptors generally—including those governing micturition—alongside the sedative and other dose-related effects discussed elsewhere in this review. In patients with unrecognised renal impairment, new or worsening urinary retention should therefore be considered alongside sedation and confusion as a possible early sign of accumulating drug exposure, rather than assumed to be unrelated to dosing.

## 12. Dose Tapering and Withdrawal: Regulatory Guidance

Baclofen withdrawal is not a theoretical risk: abrupt discontinuation—whether oral or intrathecal, deliberate or accidental, as when a pump fails, a catheter fractures, or a refill is missed—can trigger a syndrome that escalates from rebound spasticity, anxiety and pruritus to hallucinations, seizures, hyperthermia, rhabdomyolysis, multi-organ failure and death within hours to a few days [93,94,95,96]. The numbers are stark: in the first nine years of intrathecal baclofen’s post-marketing use, the manufacturer received 27 withdrawal reports, of which six patients died—roughly one in four [93]; further fatalities, including one following ventricular arrhythmia and cerebral ischaemia after pump malfunction, are documented in the case-report literature [96]. Making this more dangerous still, the clinical picture routinely mimics sepsis, meningitis, autonomic dysreflexia, malignant hyperthermia, neuroleptic malignant syndrome or serotonin syndrome—a diagnostic overlap that expert guidance identifies as a major contributor to preventable morbidity and mortality [94,95]; a 2026 systematic review of 1618 patients with oral baclofen toxicity or withdrawal similarly found severe psychiatric disturbance in up to 20.6%, alongside delirium, agitation and autonomic instability, typically within 1–4 days of stopping, with rapid baclofen reinstatement consistently resolving symptoms and reinforcing it as the correct first response [80]. Most reported severe cases were judged retrospectively to have been preventable with better patient and caregiver education and contingency planning for pump or supply interruption [94], which is precisely why regulatory bodies uniformly mandate gradual tapering rather than abrupt cessation: the FDA requires only that oral dosing be “reduced slowly”, while the EU/UK SmPC specifies roughly 1–2 weeks for oral and 2–4 weeks for intrathecal discontinuation, with published clinical protocols typically reducing the oral dose by 10–20% every 2–3 days [46,71,97,98,99]. The practical lesson is simple and non-negotiable: baclofen, in any formulation, must never be stopped abruptly, and any unplanned interruption—particularly of intrathecal delivery—is a medical emergency requiring immediate reinstatement.

## 13. Drug Interactions

Baclofen’s most clinically significant interactions are pharmacodynamic rather than pharmacokinetic and arise from additive central nervous system (CNS) and cardiovascular depression rather than from effects on drug metabolism, consistent with its minimal hepatic biotransformation (Section 5.2).

Co-administration with opioids is the interaction of greatest clinical concern. Baclofen has been shown to potentiate opioid-induced analgesia and CNS depression, and a case series of patients with intrathecal baclofen pumps who also received opioids described potentially life-threatening combined respiratory and CNS depression, with a formal Drug Interaction Probability Scale assessment supporting a probable causal relationship in several cases [100]. The combination should be used cautiously, with attention to respiratory status, particularly upon treatment initiation or dose escalation of either agent.

More broadly, co-administration with other CNS depressants—benzodiazepines, gabapentinoids, sedating antihistamines, alcohol, and general anaesthetics—produces additive sedation, dizziness, and impaired psychomotor performance, with case reports of synergistic depression even with pregabalin at conventional doses [101]. Benzodiazepines deserve emphasis: acting at GABA-A while baclofen acts at GABA-B, the two produce convergent but mechanistically distinct potentiation, and even small doses can markedly suppress mental status in a baclofen-exposed patient [84]. Alcohol is the more common real-world equivalent, worsening sedation, ataxia and respiratory depression through the same convergent pathway; this carries particular weight because baclofen is itself used off-label (and, in France, on-label) for alcohol use disorder, so a meaningful subset of patients are, by definition, at risk of drinking or relapse [102]. Patients should be counselled explicitly against combining baclofen with alcohol regardless of indication. Nabiximols (Sativex^®^), frequently used alongside baclofen, carries a labelled theoretical additive fall risk not seen in its own trials [103]; case reports show a mixed picture—one describing a supra-additive benefit allowing intrathecal dose reduction [104] and another describing new neuropsychiatric symptoms including suicidal ideation after nabiximols was added, though causality was unclear given concomitant corticosteroid use [105]. Antihypertensives, particularly vasodilators and alpha-blockers, can produce additive hypotension given baclofen’s own mild hypotensive activity [106]. Tricyclic antidepressants have been reported to potentiate baclofen’s sedative and muscle-relaxant effects. NSAIDs are a less appreciated interaction: because baclofen elimination depends almost entirely on glomerular filtration, concurrent NSAID use can reduce renal clearance enough to precipitate encephalopathy even at unremarkable doses, as in one reported case with concurrent ibuprofen and no prior renal impairment [107]. Any drug or condition reducing renal clearance will prolong baclofen’s half-life and compound the risks above (Section 14.1). Table 7 summarises the principal interactions and their management.

## 14. Use in Special Populations

### 14.1. Renal Impairment

Because baclofen is eliminated predominantly by glomerular filtration (Section 5.2), renal function is the single most important determinant of dosing outside the general titration principles described in Section 6.1. A dedicated pharmacokinetic study in patients with chronic kidney disease (CKD) found that clearance fell, and half-life and exposure rose, in proportion to renal impairment severity, proposing empirical dose reductions of roughly one-third, one-half, and two-thirds of the standard dose for mild, moderate, and severe CKD, respectively (creatinine clearance 50–80, 30–50, and <30 mL/min, respectively) [108]. Neither the FDA nor EU/UK labelling currently provides a formal renal dose-adjustment table, and case reports of baclofen neurotoxicity—including myoclonus—in patients with unrecognised renal impairment are not uncommon, sometimes on ostensibly stable, conventional-dose regimens [108]. Older patients with reduced renal function have specifically been flagged as being at elevated risk, which prompted one study to argue that baclofen warrants consideration alongside other skeletal muscle relaxants on lists of medications requiring particular caution in this group [109].

### 14.2. Pregnancy and Lactation

Baclofen crosses the placenta. UK/EU product labelling advises that, especially in the first trimester, it should be used in pregnancy only where clearly necessary, weighing maternal benefit against potential foetal risk; drug withdrawal reactions, including neonatal convulsions, have been reported after intrauterine exposure, and gradual tapering before delivery (where clinically feasible) is recommended to reduce this risk [110]. Baclofen passes into breast milk in small quantities; at therapeutic maternal doses, no adverse effects in the breastfed infant are expected, though clinical monitoring is prudent [110].

### 14.3. Older Adults

Older patients are more likely to have reduced renal function even with a normal serum creatinine (Section 14.1) and appear clinically more sensitive to baclofen’s sedative and cognitive effects at a given plasma concentration. Dose initiation at the lower end of the standard range, slower titration, and closer monitoring for confusion, falls, and excessive sedation are prudent in this group, in line with the general renal impairment precautions above.

### 14.4. Children and Adolescents

Oral baclofen is used off-label in younger children (commonly from around 2 years of age) and is labelled for spasticity from age 12 in some jurisdictions; typical paediatric regimens start at 2.5–5 mg two to three times daily, titrated to a maximum in the region of 40–60 mg/day depending on age band, with the same requirement for gradual tapering on discontinuation as in adults. Population pharmacokinetic data in children with cerebral palsy indicate a shorter mean elimination half-life (approximately 2.3–4.5 h) than typically reported in adults, with linear, weight-proportional pharmacokinetics and no clear sex difference [40]; this shorter half-life may, in principle, predispose children to more pronounced wearing-off between doses, although this has not been systematically studied specifically in paediatric MS.

## 15. Therapeutic Ratio: Animal and Clinical Data

The classical, lethality-based therapeutic index is, in fact, reassuringly wide for baclofen: the acute oral LD50 is 145 mg/kg in the rat and 200 mg/kg in the mouse [111,112], which gives a rat lethality-to-antispastic-ED50 ratio of roughly 23–50-fold (Section 5.3). This figure is mentioned for completeness, but it is a poor guide regarding the practical safety margin, because death is a terminal endpoint many-fold removed from the sedation, ataxia and CNS depression that limit dosing. A more clinically meaningful comparison—presented in Table 8—sets the effective (antispastic) dose directly against the lowest dose at which side effects appear, using the rotarod test—a well-established measure of motor coordination—in animals and, for humans, the closest available proxy to a clinical no-observed-effect level (NOEL), as no formally published regulatory NOEL for baclofen was identified. (Human overdose thresholds are discussed in Section 11 and Section 12: doses ≥ 200 mg are reliably associated with severe toxicity, and fatalities have been reported from ≥1 g [33,86,113]).

The therapeutic ratio of baclofen derived from animal and clinical data is summarised in Table 8.

Read together, the rat data make a stronger, more precise claim than the human data can, though both should be read as estimates rather than fixed biological constants. With a ratio of approximately 1, the therapeutic window in animals is, on this measure, very narrow—close to zero rather than simply reduced: the dose producing antispastic efficacy is, on average, difficult to distinguish from the dose producing detectable side effects in this model, so meaningful efficacy and some degree of motor impairment often co-occur rather than being cleanly separable by dose alone. This animal estimate is arguably the more methodologically secure of the two figures in Table 8—it comes from controlled dose–response studies with both ED50 and side-effect threshold measured on comparable functional scales—whereas the human ratio rests on a starting dose used as a proxy for a NOEL that was never formally established and plausibly overestimates the true clinical margin. The two estimates are not in direct conflict: common real-world sedation at standard doses (Section 11) is consistent with, rather than contradictory to, the animal finding, though species differences in GABA-B receptor pharmacodynamics and in the behavioural endpoints used to define a “side effect” mean that the animal ratio should not be treated as a precise, directly transferable human value. With that caveat, if a single order-of-magnitude figure is wanted for baclofen’s practical margin, the ~1 from animal pharmacology is, in the author’s interpretation, the more defensible one, and it is one of the interpretative threads this review returns to repeatedly: if efficacy and dose-limiting side effects occur at a similar systemic dose, oral dose optimisation alone has limited scope to separate them, which is part of the rationale—alongside the CSF concentration gradient discussed in Section 6.2—for intrathecal administration, concentrating baclofen at its spinal site of action at roughly 1% of the equivalent oral dose.

## 16. Discussion: Benefit–Risk Synthesis

Having reviewed baclofen’s mechanism, efficacy, long-term trajectory, adverse-effect profile, interactions, and therapeutic ratio in turn, it is worth asking the question a treating clinician and patient ultimately have to answer: taken in the round, does baclofen therapy for MS spasticity deliver a net benefit, or does it carry a net risk of harm? The evidence assembled in this review does not support a single, universal answer—it supports a conditional one, and the conditions are patient selection, route, and monitoring. Table 9 sets the two sides of this ledger against each other, domain by domain, before the discussion below weighs them explicitly.

### 16.1. The Case for Net Benefit

The benefit side of the ledger is substantial and well evidenced. Baclofen produces a real, reproducible, mechanistically well-understood reduction in spasticity across both oral and intrathecal routes (Section 7, Table 4), with intrathecal delivery achieving large relative reductions (order of 30–70%) in properly selected refractory patients. In cohorts followed for a decade or more, this benefit is generally durable rather than transient: pain, spasm frequency and satisfaction with therapy remain favourable in long-term survivors, and pharmacological treatment failure serious enough to require complete withdrawal appears uncommon (Section 10). Where oral therapy proves inadequate, intrathecal delivery offers a genuine escalation option that most other spasticity treatments cannot match in terms of magnitude of effect. Baclofen is inexpensive, generically available worldwide, reversible on stopping, and backed by fifty years of accumulated clinical experience—a track record very few alternative agents can claim (Section 8).

### 16.2. The Case for Net Harm or Meaningful Risk

Against this, the harm side of the ledger is substantial. The clinical therapeutic margin is narrow—roughly 2.5-fold between standard oral doses and doses producing severe toxicity, markedly narrower than acute-lethality-based animal data alone would predict (Section 15)—narrowing further in renal impairment, in older adults, and with common co-medications (Section 13 and Section 14). Sedation, dizziness, and cognitive slowing are common (63% vs. 36% on placebo in the original trial; Section 11), and serious neuropsychiatric, cardiovascular, and respiratory harms are documented. Withdrawal carries a real risk of death (Section 12), and real-world oral persistence is poor, with over half discontinuing within six months (Section 10)—the balance frequently resolves unfavourably outside the carefully selected, monitored patients dominating the specialist literature. Intrathecal delivery substitutes a different harm category: device-related complications occur in ~25–30% of adult patients, including infection (~6%), pump malfunction (~7%), and catheter malfunction (~14%, the leading cause of the withdrawal risk in Section 12) [51,52]. Most importantly, the one study measuring broad, validated quality-of-life outcomes found no significant improvement despite significant spasticity reduction (Section 10)—tempering any assumption that controlling the measured symptom improves what patients and clinicians ultimately care about.

The overall benefit–risk balance across the key domains discussed in this review is summarised in Table 9.

### 16.3. Weighing the Two: A Conditional Verdict

On balance, the evidence supports a net benefit for baclofen therapy in MS spasticity, but conditionally, not unconditionally. A net benefit is best supported in patients with clinically significant spasticity who are carefully selected, dosed conservatively and individually titrated, monitored for the harms detailed in this review, and—where oral therapy is inadequate—escalated to intrathecal delivery in centres experienced with its device-related complications. Here, decades of clinical experience, durable long-term control, and treatment reversibility argue for a favourable balance. Without this infrastructure, this net benefit is far less assured, and may tip towards net harm: unselected or poorly monitored oral use, unrecognised renal impairment or interacting sedatives, or any setting where withdrawal risk is not actively managed. Baclofen is therefore a genuinely effective, generally favourable therapy for the right patient when delivered and monitored appropriately—but neither a low-risk drug nor a guaranteed route to better quality of life.

### 16.4. Limitations

This review has several limitations inherent to its narrative design. First, it was not conducted as a systematic review: sources were identified through targeted rather than exhaustive, protocol-driven searching, so publication and selection bias cannot be excluded, and no formal risk-of-bias tool was applied uniformly across a very heterogeneous evidence base (randomised trials, long-term cohorts, case reports, regulatory filings, small pharmacokinetic studies, and preclinical data are all cited). Second, several quantitative estimates used for comparative purposes—including the animal-derived efficacy-to-side-effect dose ratio (Section 15), GABA-B binding affinities, and some pharmacokinetic parameters—come from single, often small, older studies rather than pooled estimates and are presented as illustrative rather than definitive. Third, direct head-to-head comparative data between baclofen and other antispastic agents, and between oral and intrathecal baclofen, remain sparse, so several conclusions rely on indirect comparison across separate study populations. Finally, the preclinical findings on glial GABA-B receptors and remyelination (Section 4.4) are drawn from rodent and ex vivo models only, with no confirmed clinical correlate in MS.

## 17. Conclusions

Baclofen remains, more than fifty years after its repurposing, the first-line pharmacological treatment for MS spasticity, with a robust evidence base and a defined place in a broader landscape including gabapentin, tizanidine, dantrolene, diazepam, nabiximols and botulinum toxin. Its principal practical limitations are pharmacokinetic and pharmacodynamic rather than a lack of antispastic efficacy: a short, variable half-life predisposing to overnight wearing-off; a narrow margin between therapeutic and toxic plasma levels, narrower than acute-lethality-based animal toxicology alone would suggest; adaptive GABA-B receptor downregulation that can necessitate planned dose holidays or, occasionally, contribute to genuine treatment failure; important interactions with opioids, alcohol, and other CNS depressants; particular caution in renal impairment, pregnancy, older adults and children; and the need for careful, gradual tapering to avoid a withdrawal syndrome that has proved fatal in reported cases. Intrathecal delivery addresses several of these limitations by concentrating the drug at its spinal site of action, at the cost of an implanted device whose complications are common rather than incidental, while newer approaches such as extended-release arbaclofen aim to improve oral dosing convenience without abandoning the same underlying GABA-B mechanism.

A further uncertainty is worth noting explicitly. The physiological consequences of continuous GABA-B receptor stimulation over months or years, as distinct from the days-to-weeks exposure captured by most of the pharmacology and toxicology literature cited here, have not been systematically characterised. GABA-B receptor downregulation (Section 9) demonstrates that the receptor system adapts to sustained agonism, but the longer-term implications of this adaptation for cognition, mood, or CNS function have not been directly studied. This sits alongside the pharmacokinetic finding in Section 15, where the best available animal evidence places baclofen’s margin between antispastic efficacy and dose-limiting toxicity at or close to unity, meaning the two doses are difficult to separate in practice. Taken together with the persistence and quality-of-life findings discussed above—poor real-world adherence and no demonstrated improvement on validated quality-of-life measures despite objective spasticity reduction—these findings may help explain why long-term persistence with baclofen is limited and why symptom-level benefits have not been shown to translate into broader patient-reported outcomes.

This review has aimed to set out the available evidence as completely as possible—covering efficacy, mechanism, pharmacokinetic constraints, the narrow therapeutic margin, receptor adaptation, and the gaps in long-term data—to support informed decision-making by patients and clinicians. On the evidence reviewed here, baclofen’s continued first-line status for MS spasticity is defensible, but the areas of remaining uncertainty identified in this review, particularly around long-term receptor-level effects, warrant consideration alongside its established efficacy when treatment decisions are made.

## 18. Future Directions

Several research priorities follow directly from the gaps identified in this review. First, arbaclofen’s confirmatory Phase 3 trial (OS440-3006), whenever it proceeds, should directly address whether its favourable tolerability profile reflects genuine stereochemical selectivity or simply the switch to twice-daily dosing (Section 6.3)—a question the existing head-to-head trial could not resolve. Second, long-term GABA-B receptor adaptation and its downstream consequences for cognition, mood, and CNS function over years to decades of continuous agonism (Section 9 and Section 17) remain essentially uncharacterised; prospective cohorts with standardised neuropsychological outcomes, rather than the retrospective and heterogeneous data available today, would meaningfully close this gap. Third, the disconnect between spasticity-specific outcome measures and broader, validated quality-of-life instruments (Section 10) warrants direct investigation: trials and registries should report both routinely, as the single study to do so found a genuine discrepancy between the two. Fourth, prospective, adequately powered, head-to-head comparisons between oral and intrathecal baclofen—rather than the indirect, cross-study comparisons this review has had to rely on—would clarify the true magnitude of benefit gained by bypassing first-pass systemic exposure. Finally, continued refinement of intrathecal pump and catheter technology, and of catheter-tip placement strategies informed by the concentration gradients described in Section 6.2, could plausibly reduce the device-related complication rate that remains intrathecal therapy’s principal practical drawback.

## Figures and Tables

**Figure 1:**
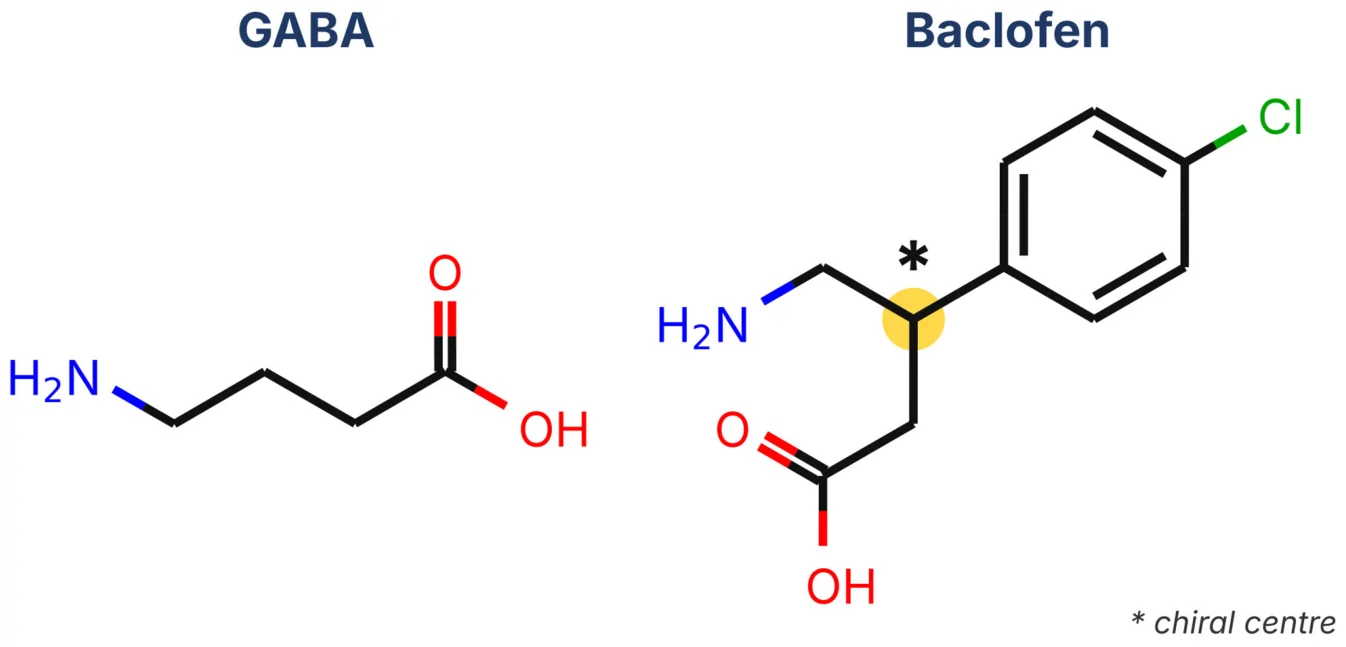
Chemical structures of γ-aminobutyric acid (GABA, 4-aminobutanoic acid, C_4_H_9_NO_2_, MW 103.1) and baclofen (4-amino-3-(4-chlorophenyl)butanoic acid, C_10_H_12_ClNO_2_, MW 213.7). Baclofen retains GABA’s full four-carbon amino acid backbone (amine → CH_2_ → CH → CH_2_ → carboxylic acid) with a 4-chlorophenyl group substituted at the β-carbon—the lipophilic addition that allows a meaningful fraction of the molecule to cross the blood–brain barrier, unlike GABA itself, while retaining GABA-B receptor affinity (Section 4.1). This β-carbon (marked * and highlighted) is baclofen’s sole chiral centre, giving rise to two non-superimposable mirror-image forms, R- and S-baclofen; marketed baclofen is the racemic (50:50) mixture of both. Section 4.1 covers their differing GABA-B receptor affinity, and Section 6.3 discusses arbaclofen, the isolated R-enantiomer developed as a purified alternative to the racemate. Structures rendered in RDKit v2026.03.

**Figure 2:**
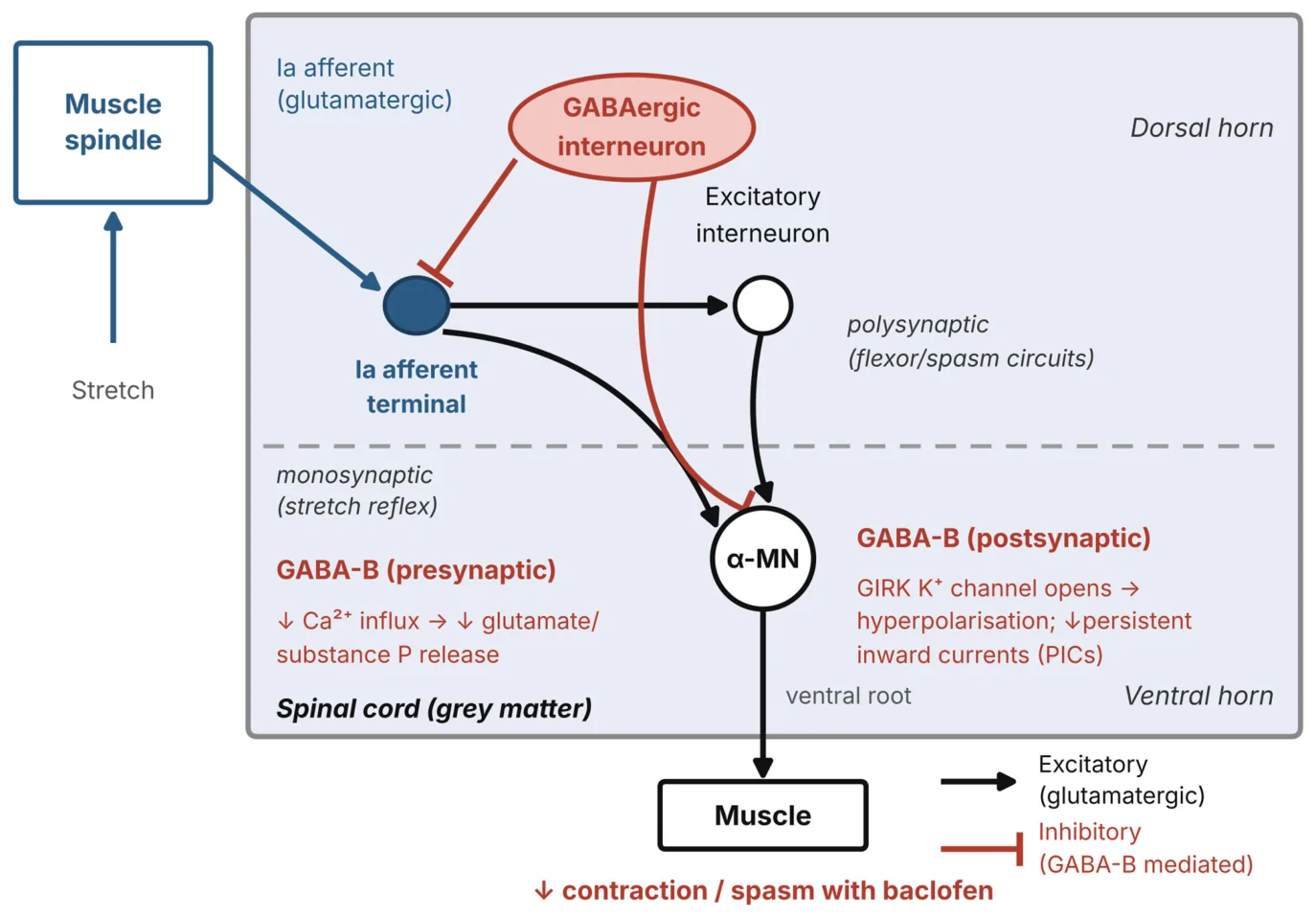
Schematic of the spinal stretch reflex arc showing baclofen’s dual GABA-B site of action. Elements shown: muscle spindle (**top left**) generating a stretch-activated Ia afferent signal; the Ia afferent terminal in the spinal dorsal horn; a GABAergic inhibitory interneuron; an excitatory (glutamatergic) interneuron mediating the polysynaptic pathway; the alpha motoneuron (α-MN) in the ventral horn; and the muscle it innervates via the ventral root. Black arrows denote excitatory (glutamatergic) connections; red lines ending in a bar denote inhibitory (GABA-B-mediated) connections. Presynaptic GABA-B receptors on the Ia afferent terminal reduce voltage-gated Ca^2+^ influx and, hence, glutamate/substance P release onto the α-MN and excitatory interneurons [21,22]. Postsynaptic GABA-B receptors on the α-MN open GIRK K^+^ channels, hyperpolarising the cell and suppressing both the monosynaptic stretch reflex and the persistent inward currents (PICs) that sustain spastic motoneuron firing [23]. Together, these two independent sites of action underlie baclofen’s efficacy against both tonic hypertonia and phasic spasms/clonus. Schematic based on the mechanisms described in Section 4.2, prepared in Matplotlib v3.10; not to anatomical scale.

**Table 1:** Key regulatory approvals of baclofen for spasticity by agency and formulation. Dates for the earliest European and UK approvals are approximate and reconstructed from historical review sources rather than primary regulatory filings, as original 1970s licensing records are not readily available online.

Agency/Region	Product/Route	Approval	Notes
USA (FDA)	Oral tablets (Lioresal^®^, NDA 020075)	1977	First approval for spasticity due to MS, spinal cord injury, and other spinal cord disease
USA (FDA)	Intrathecal (Lioresal^®^ Intrathecal)	1984 [7]	Delivered via implanted programmable pump
Europe	Oral (Lioresal^®^)	~1972 [7]	Registered nation-by-nation by Ciba-Geigy; predates the EMA (established 1995)—baclofen has never had a single centralised EU marketing authorisation, consistent with its status as a long-genericised small molecule
United Kingdom	Oral (Lioresal^®^)	~1971–1972	Marketed shortly after European registration; now regulated by the MHRA, with numerous generic SmPCs currently authorised (e.g., Accord, Advanz, Mylan/Viatris)
Switzerland (Swissmedic)/Japan (PMDA)	Oral/intrathecal	Approved (exact dates not confirmed from sources consulted)	Cited as approving agencies in WHO Essential Medicines documentation; baclofen is included in the national essential medicines lists of at least 46 countries [9]
USA (FDA)	Intrathecal injection (Gablofen^®^)	2010 [10]	CNS Therapeutics (later Mallinckrodt)
USA (FDA)	Oral granules (Lyvispah™)	2021 [11]	Saol Therapeutics

**Table 2:** GABA-B receptor location, pre-/postsynaptic site of action, and clinical correlate. The spinal sites (dorsal and ventral horn) mediate baclofen’s therapeutic antispastic effect; the supraspinal sites, present at high density throughout the neuraxis, account mechanistically for the drug’s principal dose-limiting adverse effects (Section 11) rather than representing a distinct off-target receptor mechanism.

Location	Pre/Postsynaptic	Functional Effect of Activation	Clinical Correlate
Spinal dorsal horn—central terminals of Ia and other primary afferents [21,22]	Presynaptic	Inhibits voltage-gated Ca^2+^ channels → reduced glutamate/substance P release onto interneurons and motoneurons	Reduced excitatory drive into the stretch reflex arc—core antispastic mechanism, targeted directly and preferentially by intrathecal delivery
Spinal ventral horn—interneurons and alpha motoneurons [23]	Postsynaptic	Opens GIRK/Kir3 channels → membrane hyperpolarisation → reduced firing and suppression of persistent inward currents	Suppression of monosynaptic reflex and spastic motoneuron discharge—second, independent antispastic mechanism
Cerebellum—Purkinje cell dendritic spines [17,25]	Postsynaptic (predominant)	GIRK-mediated hyperpolarisation → reduced Purkinje cell firing	Ataxia; dose-dependent rotarod impairment in animals at doses close to the antispastic range (see Section 15)
Thalamus and neocortex [24]	Both	Widespread inhibition of thalamocortical and cortical circuits	Sedation, drowsiness, cognitive slowing
Hippocampus [24]	Both	Modulation of excitatory synaptic transmission and NMDA receptor Ca^2+^ signalling	Confusion, memory impairment
Brainstem nuclei, incl. monoaminergic and autonomic control regions [24]	Both	Modulation of autonomic and monoaminergic output	Bradycardia, hypotension, dose-dependent respiratory depression in toxicity

**Table 3:** Pharmacokinetic and dose-margin evidence for baclofen, separated by whether it derives from animal/preclinical or human/clinical studies to avoid direct cross-species extrapolation. Where a parameter has not been directly studied, using comparable methodology, in the other species, this is stated rather than inferred.

Parameter	Animal/Preclinical Evidence	Human/Clinical Evidence	Ref(s)
Oral bioavailability	Not systematically reviewed for this subsection	~70–85% (healthy-volunteer crossover studies)	[32,33,34,35]
Brain/CSF vs. plasma distribution	Rat hippocampal microdialysis: ISF, whole-brain and CSF concentrations ~30-fold lower than concentration in unbound plasma at pseudo-steady state	Not measured by microdialysis in humans; low CNS penetration inferred indirectly from the systemic-dose requirement and from the intrathecal dose-sparing seen clinically (Section 6.2)	[37]
Blood–brain barrier transport mechanism	CLin ~40-fold lower than CLout in rats; efflux attenuated by probenecid, indicating transporter-mediated efflux rather than a passive permeability limit	Not directly characterised in humans; consistent in direction with the animal mechanism but not independently confirmed	[37]
Elimination half-life	Not the focus of the studies cited in this review	~2.5–6 h typically, 2.3–3.4 h in children, up to 34–36 h in overdose	[33,35,36,40,41]
Protein binding/volume of distribution	Not reviewed here	~30% protein-bound; apparent Vd ~0.7–2.5 L/kg	[36]
Renal elimination	Not reviewed here	~69% of an oral dose recovered unchanged in urine; renal clearance approximates creatinine clearance	[41]
Efficacy-to-side-effect dose ratio (therapeutic margin)	~1 (ED50 for antispastic efficacy vs. ED50 for side effects, rat; Section 15)	Classical lethal-dose-based index much wider; the narrower practical margin is inferred indirectly from clinical sedation rates rather than measured the same way as in animals (Section 15)	[see Section 15]

**Table 4:** Selected clinical efficacy data for baclofen in MS-related spasticity, oral and intrathecal. Percent changes are calculated from reported mean baseline/endpoint values where not stated directly by the source; “NR” indicates a value not reported in the source. DBPC = double-blind placebo-controlled; VAS/NRS = visual analogue/numeric rating scale.

Study	Route	N	Outcome Measure	Change	*p*-Value
Sachais et al. 1977 [61]—5-week multicentre DBPC trial	Oral (up to 70–80 mg/day)	106	Composite spasticity/spasm assessment (clinician + patient)	Significant reduction vs. placebo (exact % not reported)	*p* < 0.001
Penn & Kroin 1989 [48]—DB crossover, MS/spinal cord injury	Intrathecal (3-day infusion)	20	Ashworth score (rigidity), 0–4	4.0 → 1.2 (−70%)	*p* < 0.0001
Sammaraiee et al. 2019 [63]—20-year single-centre cohort	Intrathecal (pump, long-term)	106	Ashworth (0–4), Penn (0–4) and VAS/NRS (0–10) scores	Sustained improvement vs. baseline; 69% off oral antispastics at 1 year	*p* < 0.001
Abbatemarco et al. 2021 [64]—single-centre ambulatory MS cohort	Intrathecal (pump), 5-yr follow-up	170 implanted (256 screened)	Modified Ashworth Scale, summed across 6 muscle groups bilaterally (aggregate score range 0–30; single-joint MAS itself is 0–4)	13.5 → 4.5 (−66%) at 5 years	*p* < 0.001
Systematic review, Neurosurg Focus 2024 [65] (9 pooled ITB studies)	Intrathecal (pump), pooled	Pooled (9 studies)	Patient-Specific Functional Scale (PSFS), 0–10	Average −62.6% across studies	Significant in 7/9 studies

**Table 5:** Oral and adjunct pharmacological comparators for baclofen in MS-related spasticity: mechanism, role in therapy, and major limitations, as discussed in Section 8. Conclusions on comparative effectiveness rely on the systematic reviews and guideline sources cited, not on mechanistic reasoning alone.

Treatment	Mechanism	Role in Therapy	Major Limitations	Ref(s)
Baclofen	GABA-B receptor agonist (pre- and postsynaptic spinal inhibition)	First-line oral agent (with gabapentin); escalation to intrathecal delivery for severe/refractory or generalised spasticity	Narrow systemic efficacy-to-side-effect margin; short half-life; tolerance/withdrawal risk (Section 5, Section 9 and Section 12)	[1,2,46]
Gabapentin	Binds α2δ subunit of voltage-gated calcium channels, reducing excitatory neurotransmitter release	Co-first-line oral agent with baclofen per NICE; alternative when baclofen not tolerated	Evidence individually limited; comparative superiority over baclofen not established	[46]
Tizanidine	Central α2-adrenergic agonist, reducing excitatory input to spinal motoneurons	Second-line oral option	Sedation; hepatotoxicity risk requiring monitoring	[66]
Dantrolene	Peripheral: inhibits ryanodine receptor-mediated calcium release from sarcoplasmic reticulum in skeletal muscle	Option when a non-CNS mechanism is preferred	Hepatotoxicity; the only one of the four not acting on the CNS, so may be less effective for centrally mediated spasm	[66]
Diazepam	Positive allosteric modulator at ionotropic GABA-A receptor	Adjunct, particularly for nocturnal spasms	Sedation; dependence risk with prolonged use; pharmacodynamic interaction with baclofen (Section 13)	[66]
Nabiximols	THC/CBD oromucosal spray acting via cannabinoid receptors	Licensed add-on for spasticity inadequately controlled by other antispasticity medication, including baclofen	Observational rather than robust RCT evidence base for the add-on setting; licensed in some but not all jurisdictions	[67]
Botulinum toxin	Presynaptic inhibition of acetylcholine release at the neuromuscular junction	Focal spasticity (e.g., isolated adductor or plantarflexor spasticity); complementary to, not a replacement for, baclofen in generalised spasticity	Not appropriate for generalised spasticity; requires repeat injection	[46]

**Table 6:** Long-term (multi-year) outcome studies of intrathecal baclofen, summarising dose trajectory/tolerance and patient-reported or quality-of-life findings where reported. “Not reported” indicates that the outcome was outside that study’s scope, not that it was measured and found absent.

Study (Follow-Up)	N	Dose Trajectory/Tolerance	Patient-Reported/QoL Outcome
Heetla et al. 2009 [72] (up to 10 yr, mean 38 mo)	37	Rises then plateaus ~350 µg/day by 18 mo; tolerance in 22%	Not reported
10-year dose-by-aetiology study [73] (10 yr)	21 (MS)	83 → 227 (1 yr) → 321 µg/day (10 yr)—~4-fold rise	Not reported
7–10-year MS/SCI cohort [74] (≥7 yr)	22 (MS)	Mean dose +157–194% vs. initial optimised dose	Not reported
Mathur et al. 2014 [77] (>10 yr)	Not reported	Life satisfaction correlated positively with dose (r = 0.55, *p* = 0.02)	Low pain, normal sleepiness, moderate life satisfaction, high ITB satisfaction (8.8/10)
Prospective multicentre cohort [78] (multi-year)	115	Not the focus of this analysis	Significant spasticity reduction; EQ-5D, SIP-68 and FIM improvements small and non-significant
Ashworth-capped Japanese cohort [76] (median 6 yr 11 mo)	34	Dose increases stopped once Ashworth reached 1; tolerance in 5.9%	Ashworth stable (1.38 → 1.39) on a comparatively low, stable dose

**Table 7:** Principal clinically significant drug interactions with baclofen and their management. Interactions are predominantly pharmacodynamic (additive CNS/respiratory/cardiovascular depression) or related to reduced renal clearance, consistent with baclofen’s minimal hepatic metabolism.

Interacting Agent/Class	Nature of Interaction	Clinical Management
Opioids [100]	Potentiation of analgesia and combined CNS/respiratory depression; a case series links concomitant intrathecal baclofen and opioids to potentially life-threatening respiratory depression.	Use lowest effective doses; monitor respiratory status closely at initiation or dose changes.
Benzodiazepines/Z-drugs [84]	Convergent GABAergic potentiation (GABA-A + GABA-B); even small doses can markedly suppress mental status.	Avoid where possible; if combined, use conservative dosing and enhanced monitoring.
Alcohol [102]	Convergent GABA-A + GABA-B potentiation causing severe sedation, ataxia and respiratory depression; relevant because baclofen is also used off-label (on-label in France) for alcohol use disorder.	Counsel explicitly against alcohol use regardless of indication; use structured titration and monitoring when treating alcohol use disorder specifically.
Nabiximols (Sativex) [103,104,105]	UK/EU labelling flags a theoretical additive fall risk not seen in Sativex trials; case reports show a mixed picture—one reporting supra-additive benefit allowing dose reduction, another reporting new neuropsychiatric symptoms including suicidal ideation (causality unclear).	Warn of theoretical additive sedation/fall risk; monitor mood after starting; a reasonable trial in refractory spasticity with the same caution as other CNS-active combinations.
Other CNS depressants (sedating antihistamines, gabapentinoids, general anaesthetics) [101]	Additive sedation, dizziness, and psychomotor impairment; case reports of synergistic CNS depression with pregabalin at conventional doses.	Use cautious co-titration with gabapentinoids or other sedatives.
Antihypertensives, vasodilators, alpha-blockers [106]	Additive hypotensive/orthostatic effects.	Monitor blood pressure on initiation/dose changes; counsel on postural hypotension.
Tricyclic antidepressants	Case-level evidence of potentiated muscle relaxation and sedation, occasionally with pronounced hypotonia.	Dose caution rather than avoidance; monitor for excessive weakness or sedation.
NSAIDs [107]	Reduced renal clearance of baclofen (both agents affect/depend on renal function), reported to precipitate encephalopathy even at low baclofen doses in a patient without known renal impairment.	Be alert to toxicity with concurrent regular NSAID use, particularly in older patients or those with borderline renal function.
Antipsychotics/neuroleptics	May lower seizure threshold and can potentiate CNS/respiratory depression; neuroleptics are contraindicated in patients with severe CNS depression.	Use with caution; avoid in comatose or severely CNS-depressed patients.

**Table 8:** Therapeutic ratio of baclofen derived from a direct comparison of efficacy dose against side-effect dose (animal) or the best available clinical NOEL proxy (human) rather than against acute lethality. Both rows point to the same conclusion reached qualitatively elsewhere in this review: baclofen’s practical margin between benefit and harm is narrow and considerably narrower than a classical LD50-based therapeutic index would suggest.

Basis	Efficacy Dose	Side-Effect Dose/Clinical NOEL	Ratio	Notes
Rat, antispastic pharmacology vs. motor side effects [114,115]	ED50 ~ 2.9 mg/kg i.d. (sustained decerebrate forelimb rigidity); ED50 ~ 6.2 mg/kg (phasic rigidity)	Rotarod-detectable ataxia reported from ~3 mg/kg PO, dose-dependent and markedly strain-dependent; no formal TD50 established in the literature reviewed	~1.0–1.1 (sustained)/~0.5 (phasic)	Side-effect and antispastic doses essentially coincide, or side effects appear at a lower dose than full phasic efficacy—in sharp contrast to the wide ~23–50-fold margin against acute lethality (see text)
Human, therapeutic dose vs. clinical NOEL [33,61,62]	Standard effective oral range: 30–80 mg/day (FDA maximum 80 mg/day)	No formally published regulatory NOEL/NOAEL for baclofen was identified; the lowest standard starting dose (5 mg) is used here as the best available practical proxy, though this likely overstates true tolerability—sedation is reported in the majority of patients (63% vs. 36% on placebo) even within the standard effective dose range itself	~6–16 (80 mg/5 mg, practical proxy only)	This wider ratio is an artefact of using the lowest tolerated starting dose as a stand-in NOEL; because adverse effects are common within, not only above, the therapeutic range, it should not be read as a genuine clinical safety margin—the rat row is the more honest picture of baclofen’s practical margin

**Table 9:** Benefit–risk balance across key domains discussed in this review. “Verdict” reflects the weight of evidence for a well-selected, individually titrated, and actively monitored patient; it is not a claim that every domain is favourable for every patient, and several rows (withdrawal risk, real-world oral persistence, device complications, quality of life) argue for caution even in this best-case framing.

Domain	Evidence Favouring Benefit	Evidence Favouring Harm/Caution	Verdict
Antispastic efficacy	Robust, reproducible, mechanistically grounded; large effect sizes with ITB (~30–70% reduction)	Older oral trials report significance without quantified effect size	Favours benefit
Long-term durability	Sustained pain/spasm control > 10 years in survivors; true treatment failure uncommon	Continued dose escalation in some cohorts; survivorship bias likely inflates the reassuring picture	Favours benefit, with caveat
Therapeutic margin	Wide margin vs. acute lethality in animals (~23–50-fold)	Narrow clinical margin (~2.5-fold); narrows further with renal impairment, age, and interacting drugs	Favours caution
Common adverse effects	Generally, dose-related and reversible with dose reduction	Sedation in 63% vs. 36% on placebo; ataxia and cognitive slowing frequent	Favours caution
Serious/rare adverse effects	Individually uncommon	Suicidal ideation, psychosis, paradoxical hypertension, and dose-dependent respiratory depression all documented	Favours caution
Withdrawal risk	Manageable with gradual, individualised tapering	Case-fatality ~1 in 4 among reported post-marketing cases; symptoms mimic other emergencies, which delays recognition	Highest-stakes risk in the review
Drug interactions	Manageable with counselling and monitoring	Opioids, alcohol and benzodiazepines all carry life-threatening potential; NSAIDs and renal impairment compound risk	Favours caution
Real-world persistence (oral)	Not applicable	More than half of patients discontinue within 6 months in population-based data	Favours harm in unselected use
Intrathecal device/procedural risk	Avoids most systemic oral risks for a given antispastic effect	~25–30% of adult patients experience a device-related complication over follow-up	Favours caution
Quality of life/function	Symptom-level (spasticity) success is well documented	The one study using validated, generic QoL/functional instruments found no significant improvement	Unclear—favours caution about overclaiming
Cost, reversibility, track record	Inexpensive, generically available, fully reversible on stopping, 50 years of clinical experience	Not applicable	Favours benefit

## Data Availability

No new data were generated or analysed in this review. All data discussed are available in the cited primary sources.
